# Ethanol and Higher Alcohols’ Production in Fungal and Bacterial Laboratory Cultures and Significance for Forensic Samples

**DOI:** 10.3390/microorganisms12030462

**Published:** 2024-02-24

**Authors:** Vassiliki A. Boumba, Glykeria Velivasi, Nikolaos Kourkoumelis

**Affiliations:** 1 Departments of Forensic Medicine & Toxicology, Faculty of Medicine, School of Health Sciences, University of Ioannina, 45500 Ioannina, Greece; glykeria.velivasi@gmail.com; 2 Departments of Medical Physics, Faculty of Medicine, School of Health Sciences, University of Ioannina, 45500 Ioannina, Greece; nkourkou@uoi.gr

**Keywords:** ethanol, postmortem, higher alcohol, 1-propanol, 1-butanol, modeling, blood, forensic toxicology

## Abstract

Ethanol can be produced by many microorganisms that colonize a dead body. Ethanol’s concentration depends on the congener higher alcohols, 1-propanol, isobutanol, 2-methyl-1-butanol, 3-methyl-1-butanol, and 1-butanol, as previous research has shown. This correlation is expressed by mathematical models which estimate the concentration of microbial ethanol. The aim of this contribution was to study the ethanol and higher alcohols’ production in various laboratory bacterial and fungal cultures and the applicability of the bacterial and fungal models (which concern the bacteria *E. coli*, *S. aureus*, *K. pneumoniae*, and *E. faecalis*, and the fungus *C. albicans*) in these samples, as well as in blood samples from autopsy cases, with the overall objective of investigating the models’ applicability in routine casework. The bacteria and fungus were cultured in conventional culture media and in denatured human blood cultures under various conditions. The alcohols’ concentrations were determined using a head space–gas chromatography–flame ionization detector (HS-GC-FID). The previously reported bacterial and yeast models were applied in the cultured samples and in blood from 122 autopsy cases. Our results showed that 1-propanol was not produced by *C. albicans* and *E. faecalis* under certain conditions. Also, 1-butanol was not produced by *C. albicans*, *E. faecalis*, and *K. pneumonia* under certain conditions. Furthermore, the bacterial models were applicable in postmortem samples irrespective of the microbes that were possibly activated in the sample, while the EC models showed the best applicability among all the bacterial and yeast models. The best applicability of the bacterial models was observed in autopsy blood with 0.10 g/L < BAC < 1.0 g/L in cases of violent and undetermined causes of death and in cases with putrefaction. Finally, the yeast models were applicable in limited, possibly special, autopsy cases. In conclusion, it could be inferred that the source of ethanol in any given postmortem blood sample is likely microbial if either most bacterial models or at least one model from each distinct bacterial species is successfully applicable.

## 1. Introduction

Ethanol (“alcohol”) consumption (“drinking”) is prevalent worldwide and related to a wide set of adverse health, social, and economic consequences [1]. The major problems of excessive drinking and drunkenness have an impact on law enforcement, since people under the influence of alcohol commit various crimes, such as drunken driving, sexual assault, domestic violence, or even murder [2]. Therefore, assessing alcohol consumption is crucial for the forensic investigations of accidents, crimes, or deaths [2,3]. Blood is the preferred specimen for ethanol analysis, and the relative legislation sets legal limits for alcohol consumption according to blood alcohol concentration (BAC) [3]. The measurement of BAC is the simplest and most frequently performed assay in a forensic toxicology laboratory while the evaluation of the relevant toxicological results is often part of the judicial inquiry in criminal or civil courts [3,4]. The evaluation of the analytical data is used to support the forensic evidence on the interrelation between the consumption of alcoholic beverages, the measured BAC, and the impairment of body functions [4]. Particularly in postmortem cases, BAC is determined as part of the death investigation process since it might be a causal or a contributory factor [3,4]. In such cases, the interpretation of relative results may present limitations and complexities mainly due to the postmortem ethanol neo-formation (which is actually microbial ethanol production) [5,6,7,8,9] at the interval from death up to the analysis of the sample. To achieve a feasible accuracy in interpreting the postmortem ethanol analysis results and in defining the origin (whether it is a result of antemortem consumption or postmortem microbial production or both) of the measured ethanol, various factors have been evaluated [5,10,11], such as the putrefaction state of the cadaver at autopsy, the clinical history of the deceased, the determination of non-oxidative metabolites of ethanol, the identification of microbes in the analyzed sample, and the evaluation of the discrepancies between ethanol concentration from various sampling sites and from different specimens [5,12,13,14,15,16,17]. Furthermore, the detection of low-molecular-weight volatiles in postmortem specimens was related to the microbial ethanol neo-formation [18,19,20,21,22,23]. Specifically, the volatile compounds known as higher, or congener, alcohols—referring mainly to 1-propanol (or n-propanol), isobutanol (or methyl-1-propanol), 2- methyl-1-butanol (or isoamyl-alcohol), 3-methyl-2-butanol (or amyl-alcohol), and 1-butanol (or n-butanol)—are often detected in postmortem specimens [24,25,26], and their presence is considered indicative of putrefaction and microbial activity [18,27]. Moreover, relatively recently, we reported the process characterized as “modelling postmortem ethanol production” [28,29,30,31] that introduced the models constructed by performing experimental studies with the bacteria *E. coli* [28,29], *K. pneumoniae* [30], *S. aureus* [30], *E. faecalis* [30], the clostridia *C. perfringens* [28], and *C. sporogenes* [28], and the fungus *C. albicans* [31]. The models are parsimonious first-order mathematical equations, where ethanol is the dependent variable, and the higher alcohols are the independent ones, and they allow for the calculation of the microbially produced ethanol in various samples where at least one higher alcohol has been detected simultaneously with ethanol, irrespective of which microbes have been activated in the sample. Overall, the “modelling” of the postmortem ethanol production with the relevant models suggests that the higher alcohols are quantitative biomarkers of microbial ethanol production and should be monitored in postmortem cases.

The objective of this contribution was to record and compare, firstly, the ethanol and higher alcohols’ concentrations produced in various laboratory bacterial and fungal cultures, different from those used to construct the models, and, secondly, to test the applicability of the previously reported bacterial (*E. coli*, *S. aureus*, *K. pneumoniae*, and *E. faecalis*) and fungal (*C. albicans*) models in samples from respective microbial cultures in conventional culture media and human denatured blood, as well as in blood from real autopsy cases. As the applicability of the models means their potential to accurately calculate the concentration of the microbially generated ethanol from the concentrations of the congener alcohols in the sample, the overall aim was to investigate the models’ applicability in routine casework.

## 2. Materials and Methods

### 2.1. Microbial Cultures in Conventional Culture Media

The microbial cultures in culture media at 37 °C were developed as previously described for the bacteria [30] and fungus [31]. Briefly, each one of the cryopreserved bacteria, *Staphylococcus aureus* ATCC 25,923, *Enterococcus faecalis* ATCC 29,212, *Klebsiella pneumoniae* ATCC 13,883, and *C. albicans* strain *C. albicans* ATCC 10,231, was revived into a selective nutrient agar (Columbia Blood agar) (Becton Dickinson GmbM, Heidelberg, Germany) or Sabouraud Dextrose Agar (SDA) (Neogen, Lansing, MI, USA), respectively, and incubated at 37 °C aerobically for 24–48 h. Standard microbial suspensions of approximately 10^8^ CFU/mL with an optical density of 0.605 at a wavelength of 620 nm were prepared by transferring 5–6 colonies of the initial culture into sterile saline. Sets of 16 sterile polystyrene tubes (Aptaca, Canelli, Italy) containing 5 mL of sterile Brain Heart Infusion (BHI) broth (Biolife Italiana S.r.l., Milan, Italy) for each bacteria and the fungus and sterile Sabouraud Dextrose Broth (SDB) (Sigma-Aldrich, Darmstadt, Germany) for the fungus were inoculated with 50 μL of each microbial suspension separately to obtain a final concentration of 10^6^ CFU/mL. The pH of the BHI medium for the fungal cultures was adjusted to 5.6, equal to that of SDB. One milliliter of sterile paraffin oil was added in each tube followed by anaerobic incubation (An conditions) into “anaerobic jars” (GasPak System, 45534, Biomeriuex, France) at 37 °C for 7 days. In parallel experiments, the bacterial and fungus species were cultured aerobically for the first 4 days, and then 1 mL of sterile paraffin oil was added to each tube, and the tubes were placed into anaerobic jars to provide anaerobic conditions from day 4 to day 7 at 37 °C (AA conditions). The fungus was cultured in two different culture media, Brain Heart Infusion broth (BHI) and Sabouraud Dextrose broth (SDB), separately under aerobic/anaerobic (AA) and strictly anaerobic incubation (An) in acidic and neutral cultures, while the bacteria were cultured only in BHI under both aeration conditions.

### 2.2. Microbial Cultures in Human Denatured Blood

Bacterial and fungal cultures in human denatured blood (hDB) were developed as previously described [30,31]. Human whole blood was pooled from healthy individuals whose blood glucose levels ranged between 0.9 and 1.3 g/L, placed in a sterilized glass bottle, and heated at 50 °C for about two hours, with gentle stirring to denature plasma proteins. The pH of the denatured blood was 7.2 ± 0.2 compared to 7.4 ± 0.2 of the original blood. Two milliliter portions of denatured blood were transferred into sterilized glass tubes (16 tubes per different culture). Each tube was inoculated with the appropriate volume of bacterial suspension in order to obtain an initial concentration of 10^6^ CFU/mL (equal to the culture concentrations used to build the relevant microbial models). Samples were incubated at 25 °C under aerobic/anaerobic (AA) conditions by adding a sterile paraffin oil overlay on the 4th day of incubation and under strictly anaerobic (An) conditions in respective sets of samples in parallel. *C. albicans* was cultured in both neutral (neu) and acidic (ac, acidified with HCl 5 M) hDB, with or without the addition of either a sterile, concentrated glucose solution of 200 g/L (Merck KGaA, Darmstadt, Germany) so as to obtain 0, 1, 3, 7, and 11 g/L additional glucose concentrations, respectively, or diluted hDB (1:5 *v*/*v*) with sterile dextrose (dxtr) 5% *w*/*v* solutions. All samples were incubated at 25 °C for 7 days under AA conditions. All the aforementioned tests in denatured human blood were performed in parallel.

### 2.3. Alcohols’ Measurement

On days 0, 1, 2, 3, 4, 5, 6, and 7, two tubes from each set of samples/different cultures were removed from the incubation system, and the alcohols’ concentrations were determined using HS-GC-FID [30,31]. The higher alcohols 3-methyl-1-butanol and 2-methyl-1-butanol are reported as methyl-butanol (sum of the two isomers) for consistency with our previous studies.

### 2.4. Autopsy Cases

Our chromatograms’ archives were retrospectively reviewed, and those having BAC > 0.10 g/L and detectable concentrations of at least one higher alcohol were selected for further evaluation. A total of 122 chromatograms from respective postmortem blood samples corresponding to respective autopsy cases were selected and evaluated in regard to the BAC levels, the manner of death, and the putrefaction state of the corpse at autopsy.

### 2.5. Models

The previously developed bacterial [28,29,30] and fungal [31] models (presented in Table 1) were used to calculate the theoretical microbial ethanol produced in the samples from laboratory cultures and from autopsy cases. The abbreviations EC1-7 stand for the seven *E. coli* models [28,29], EF for the single *E. faecalis* model [30], KP1-4 for the four *K. pneumoniae* models [30], SA1-2 for the two *S. aureus* models [30], and CA1-7 for the seven *C. albicans* models [31].

## 3. Results

### 3.1. Comparison of Ethanol and Higher Alcohols in Laboratory Microbial Cultures

In Table 2 are presented the ranges of ethanol and higher alcohol concentrations produced in the microbial cultures of conventional culture media (BHI and SDB) at 37 °C. For all sets of samples, alcohols were produced on the first 4 days of incubation, and then they remained rather stable. Ethanol concentrations predominated among the alcohols’ concentrations and were comparable in the cultures of the same microbe (bacterium or fungus) cultivated in the presence or absence of air (Table 1). *K. pneumoniae* produced more ethanol than the other bacteria under the applied conditions. Higher alcohol concentrations were comparable in the cultures of *E. faecalis*, *S. aureus*, and *C. albicans* cultivated in the presence or absence of air, but *K. pneumoniae* produced more 1-propanol under aerobic conditions than under mixed aerobic/anaerobic conditions. The most abundant higher alcohol was 1-propanol for *K. pneumoniae*, 1-butanol for *E. faecalis*, and methyl-butanol for *C. albicans*. As regards the SDB *C. albicans* cultures, more ethanol and higher alcohols were produced than in the BHI cultures. Interestingly, 1-butanol was not produced in any set of fungus cultures.

The patterns of ethanol and higher alcohols produced in the hDB microbial cultures at 25 °C (shown in Table 3) were variable. It can be noticed that hDB was used as a substitute of postmortem blood. All tested bacteria produced comparable ethanol levels, except *E. faecalis*. Moreover, *E. faecalis* produced only methyl-butanols. Furthermore, *K. pneumoniae* did not produce 1-butanol and isobutanol. It is worth underlining that only *S. aureus*, among the tested microbes, produced all higher alcohols.

Ethanol concentrations of up to 4.7 g/L were produced in the hDB fungal cultures, and they were proportional to the concentration of the added glucose. Also, only methyl-butanols were produced in the hDB cultures without additional glucose. Finally, 1-butanol was not produced in any set of fungal cultures.

### 3.2. Comparison of the Application of Bacterial and Fungal Models in Laboratory Microbial Cultures

The applicability of each one of the models, described by equations 1–21 (Table 1), was tested for the samples from the microbial cultures developed in conventional culture media and those in hDB. The samples with ethanol higher than 0.10 g/L were used for the evaluation of the applicability of the models. The theoretical microbial ethanol was calculated by using the models, and the received value was compared to the respective measured ethanol concentration. A standard error (E%) less than ±40% was set as the cut-off for considering the application of each model successful when comparing the calculated ethanol concentration to the measured ethanol concentration. The numbers of successive predictions for the cultured samples in conventional culture media and those in hDB are shown in Table 4 and Table 5, respectively.

As can be seen in Table 4, the bacterial models were applicable in more samples from the conventional bacterial cultures than from the respective fungal cultures. Specifically, the models EC1–4, EC6, and KP1 were applicable in both bacterial and fungal samples. The fungal models were applicable only to fungal samples, while the models CA1–3 were applicable in more samples than the other fungal models.

As can be seen in Table 5, the bacterial models were better applicable than the fungal ones in samples from different hDB cultures. More specifically, at least one of the EC and KP models was successfully applicable to samples from all the hDB bacterial and fungal cultures. At least one of the EC4–6 models achieved applicability to samples from all the different bacterial or fungal hDB cultures. Particularly, the EC4 model was applicable even in samples where 1-butanol was not produced. Interestingly, the KP1 model was applicable in fungal samples with additional glucose. Finally, the CA models were applicable mainly to samples from the fungal hDB cultures.

### 3.3. Application of Bacterial and Fungal Models in Autopsy Bloods

The applicability of the models described by equations 1–21 was tested in the selected 122 postmortem blood samples from the respective number of autopsy cases that had BAC > 0.10 g/L and at least one detectable higher alcohol. The applicability of the models was considered successful when the standard error (E%) was less than ±40% in comparing the calculated ethanol concentration versus the measured ethanol concentration for each sample (applied formula: E% = [(C_calc_ – C_measured_)/C_measured_] × 100).

**Figure 1:**
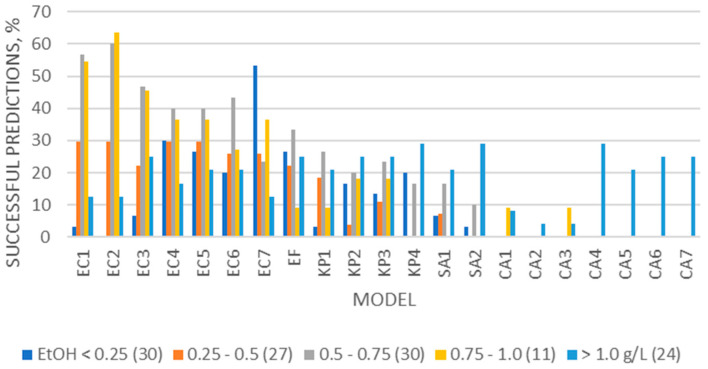
Percentages of successful predictions (having E% ≤ ±40%) for each group of cases, categorized according to the measured BAC, when each model was applied to the respective postmortem sample.

In Figure 1 are shown the “successful” cases when the respective samples were categorized according to the measured BAC. As can be seen, the bacterial models were successfully applicable in autopsy samples from all BAC categories. The EC models were the most successful of all bacterial models, while the most successful predictions were achieved for the cases with BAC from 0.50 to 0.75 and from 0.75 to 1.0 g/L. The KP2–4 and SA1–2 models were more successful for cases with BAC > 1.0 g/L. The EF model was more successful for cases with BAC < 0.75 g/L and >1 g/L. On the other hand, the fungal models were only applicable in a few cases with BAC higher than 1.0 g/L.

**Figure 2:**
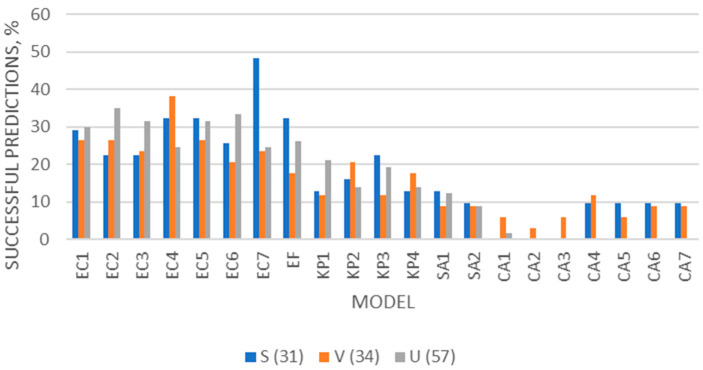
Percentages of cases categorized according to the manner of death (sudden, violent, and unspecified manner of death cases), having E% ≤ 40% when each model was applied to the respective postmortem sample.

In Figure 2 are shown the “successful” cases when the respective postmortem samples were categorized according to the manner of death. As can be seen, the bacterial models were, generally, more successful than the fungal models. All bacterial models were more successful in cases from an undetermined cause of death. The EC models were the most successful bacterial models (up to 48% of cases). The fungal models succeeded in the scores of few sudden or violent death cases.

**Figure 3:**
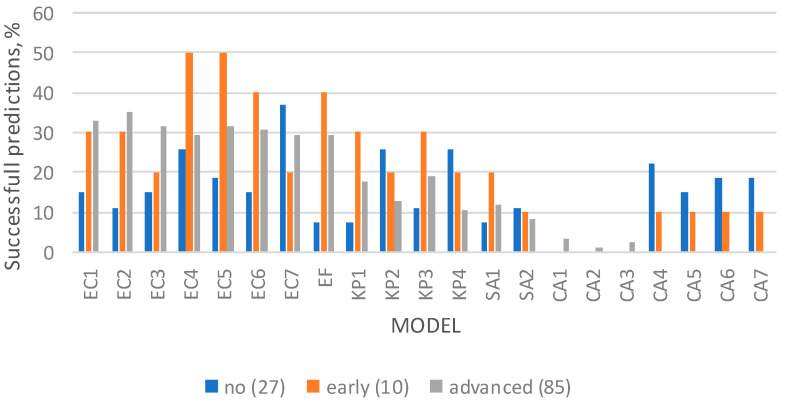
Percentages of cases categorized according to the degree of putrefaction at autopsy, having E% ≤ 40% when each model was applied to the respective postmortem sample.

In Figure 3 are shown the “successful” cases when the respective samples were categorized according to the degree of putrefaction of the corpse at autopsy. As can be seen, the bacterial models were more successful than the fungal ones. Apparently, all the bacterial models were more successful in cases with advanced or early putrefaction at autopsy than in cases with no putrefaction. The CA models were successful in limited cases.

Overall, the *E. coli* models had the best applicability of all, as they were successful in 76/122 autopsy cases (62%). The *K. pneumoniae* models were applied successfully in 40/122 cases (33%), the *E. faecalis* models in 25/122 cases (20%), and the *S. aureus* models in 18/122 cases (15%). Finally, the *C. albicans* models had the lowest applicability of all models, as they were applicable only in 10/122 cases (8%), 9 of them at the high BAC group. Also, there was a number of autopsy cases (26/122 cases, 21%) where none of the bacterial or fungal models was applied successfully.

## 4. Discussion

The decomposition of a human body post-mortem, viewed through the lens of a cynic, is a rich matrix of organic compounds, crucial for the proliferation of microbes. This system’s complexity is underscored by the variability in decomposition processes across different bodies, environmental conditions, and even within various sections of the same cadaver [32,33]. From a biochemical perspective, microbial activity in a deceased body mirrors any system containing substrates favorable to microbial growth under optimal conditions [33]. From a microbiological point of view, the presence of specific substrates directly influences microbial metabolism, which is further modulated by the intricate interplay of competitive and cooperative interactions among different microbiota species, leading to unpredictable outcomes. Such dynamic systems are challenging to replicate precisely outside of in vivo conditions and are typically approximated in controlled laboratory settings.

Experimental studies on conventional microbial cultures in BHI and SDB media at 37 °C have demonstrated distinct variations in alcohol production patterns compared to the original cultures at 25 °C [28,29,30,31]. For instance, at 37 °C, *K. pneumoniae* exhibited a decrease in 1-propanol and 1-butanol production, while increasing methyl-butanols output compared to conditions at 25 °C; *S. aureus* at 37 °C showed reduced ethanol and 1-propanol levels but an increase in isobutanol production compared to the cooler temperature. Also, slightly more 1-propanol was produced in fungal cultures at 37 °C than at 25 °C. These observations are in line with previous findings, asserting temperature’s role in modulating ethanol and higher alcohols’ synthesis [34,35] besides the type of microbe.

The hDB medium was used as a surrogate for microbe-free and alcohol-free post-mortem blood [30,31]. The denaturation process effectively disrupts the secondary and tertiary structures of cellular and biopolymer molecules, rendering hDb as an analog to postmortem blood. Optimizations for fungal growth, such as increased glucose concentrations and acidic conditions, were specifically applied to fungal cultures [31,36].

Interestingly, the non-production of 1-propanol and 1-butanol in certain microbial culture sets highlights the variability and selective nature of microbial congener alcohol synthesis, corroborating reports of their absence in postmortem cases where ethanol production was detected [18,26,37,38,39]. This variability reinforces the argument for considering the five higher alcohols (isobutanol, 2-methyl-1-butanol / amyl alcohol and 3-methyl-1-butanol / isoamyl alcohol, 1-butanol and 1-propanol) as critical biomarkers for microbial ethanol synthesis [26], advising against their use as internal standards in forensic laboratories during ethanol analysis due to potential postmortem microbial production.

Our results (presented in Table 4 and Table 5) showed that the bacterial models, and especially the EC ones, could apply in samples cultivated with different microbial species, bacteria or fungi, indicating their application potential in real cases. Regarding autopsy bloods, the EC models showed the best applicability among the bacterial and yeast models, and especially in cases with BAC ranging from 0.5 to 1.0 g/L (57–64% of samples for EC5–6 and almost 40% of samples for EC1–4) and in cases with putrefaction (20–50% of putrefactive cases) (Figure 1). These findings in autopsy bloods, when compared with the previous results on the applicability of the clostridial models that were reported to apply successfully in up to 95% of autopsy cases with obvious signs of putrefaction and up to 85% of cases with BAC < 0.7 g/L [26,28], showed that the EC models followed the clostridial ones in applicability. The fungal models were by far the least applicable of all; possibly, they can be applicable in specific cases with elevated glucose levels. The EC models were the most successful of the tested models in all manner of death cases (Figure 2), showing that the latter is not an indicative factor of postmortem ethanol production. The bacterial models were more successful in cases with putrefaction (Figure 3), as expected, since putrefaction is a well-recognized indicator of microbial ethanol production at the postmortem [5,6,8,22,24].

A thorough analysis of the applicability of various models across the 122 cases revealed that a significant majority of the bacterial models (8 out of 14) was applicable in 17 instances—comprising three sudden deaths, five violent deaths, and nine deaths of unspecified causes. Notably, the majority of these instances (15 out of 17 cases) involved bodies that exhibited either early or advanced stages of putrefaction at the time of autopsy. The ethanol concentrations in twelve of these cases fell within the range of 0.20–0.81 g/L; such levels are generally attributed to microbial production as previously reported [5,6]. Only one case had elevated ethanol concentration (3.72 g/L), but since it corresponded to a case with putrefaction, the origin of ethanol possibly could be, partially, antemortem consumption and, partially, postmortem formation. It is worth mentioning that 2 out of the 17 cases (cases 63 and 74) were violent deaths without signs of putrefaction at autopsy, indicating that microbial ethanol production could have happened before the appearance of obvious putrefaction, especially in cases with open wounds present to the corpse [27], as in these two cases; the presence of wounds to the corpse increases the possibility of a microbial invasion from the environment to the dead body, and, hence, it promotes the expansion of internal microflora [40]. Furthermore, at least one bacterial model from a different bacteria species was applied successfully to 5 out of the aforementioned 17 cases, all having early or advanced signs of putrefaction and BAC ranging from 0.16 to 0.71 g/L. Due to the characteristics of these specific five cases, it is most likely that the ethanol of these samples is of microbial origin. Therefore, it could be inferred that the source of ethanol in any given postmortem blood sample is likely microbial if either most bacterial models or at least one model from each distinct bacterial species is successfully applicable (within E ≤ ±40%). Certainly, future research is needed to investigate and elucidate the extent and the possible limitations of the models’ use in postmortem forensic practice.

In conclusion, it is recommended to apply the bacterial, clostridial, and fungal models to all “suspected” postmortem cases in which ethanol and (at least one) higher alcohol(s) are simultaneously detected to assess the ethanol concentration levels attributed to microbial production, which then, in conjunction with case-specific characteristics, would enable a more precise determination of the ethanol origin in the samples.

## 5. Conclusions

Our findings suggest that propanol and butanol are not synthesized by *C. albicans*, *E. faecalis*, and *K. pneumonia*, indicating a notable absence of these alcohols in the metabolic profiles of these organisms under specific conditions. Furthermore, the bacterial models are valid in postmortem samples, regardless of the microbes that might have been activated within the sample. These models hold significance in autopsy cases where BAC ranges from 0.10 to 1.0 g/L, as well as cases exhibiting signs of putrefaction. Among the models evaluated, those based on EC showed the best applicability of all bacterial models, albeit to a lesser extent than those based on clostridial species. Conversely, fungal models are potentially restricted to specific autopsy cases.

## Figures and Tables

**Table 1:** List of bacterial and fungal models to calculate the microbial generated ethanol concentration (in g/L) from the concentrations of the congener higher alcohols (in mg/dL). Abbreviations refer to the concentrations of alcohols as follows: ET for ethanol, PR for 1-propanol, IB for isobutanol, BU for 1-butanol, MB for methyl butanols.

	No	Model	Model (Equation)	Ref.
Bacterial	1	EC1	ET = 0.07 * PR + 0.20 * IB + 1.61 * BU + 1.15 * MB + 0.15	[16]
	2	EC2	ET = 0.08 * PR + 1.57 * BU + 1.18 * MB + 0.15	[14,16]
	3	EC3	ETl = 2.25 * BU + 0.98 * MB + 0.15	[16]
	4	EC4	ET = 0.07 * PR + 3.77 * IB − 0.26 * BU + 0.07 * MB + 0.39	[17]
	5	EC5	ET = 0.05 * PR + 1.13 * IB + 0.95 * BU − 1.60 * MB + 0.31	[17]
	6	EC6	ET = 0.05 * PR + 0.53 * BU + 0.32	[17]
	7	EC7	ET = 0.05 * PR + 1.06 * IB + 1.76 * BU − 1.62 * MB + 0.14	[17]
	8	EF	ET = 0.16 * PR − 1.24 * IB + 0.27 * BU + 0.09	[18]
	9	KP1	ET = 0.13 * PR + 6.17 * IB + 1.37 * BU − 0.43 * MB − 0.02	[18]
	10	KP2	ET = 0.31 * PR − 0.41 * MB + 0.05	[18]
	11	KP3	ET = 0.23 * PR + 1.20 * BU − 0.27 * MB + 0.03	[18]
	12	KP4	ET = 0.36 * PR − 0.71 * MB + 0.10	[18]
	13	SA1	ET = 0.28 * PR + 3.52 * IB + 0.91 * BU − 1.05 * MB	[18]
	14	SA2	ET = 0.40 * PR + 5.33 * IB + 0.04 * BU − 1.65 * MB + 0.01	[18]
Fungal	15	CA1	ET = 3.01 * PR − 0.09 * MB + 0.46 * IB + 0.28	[19]
	16	CA2	ET = 3.98 * PR − 0.25 * MB + 1.10 * IB + 0.33	[19]
	17	CA3	ET = 4.30 * PR + 0.29 * IB (or MB) + 0.28	[19]
	18	CA4	ET = 10.4 * PR − 2.24 * MB + 9.62 * IB + 0.76	[19]
	19	CA5	ET = 10.4 * PR + 5.58 * IB (or MB) + 0.43	[19]
	20	CA6	ET = 20.6 * PR + 4.13 * MB − 5.16 * IB − 0.42	[19]
	21	CA7	ET = 21.5 * PR + 1.31 * IB (or MB) − 0.38	[19]

**Table 2:** Ranges of ethanol and higher alcohols’ concentrations in conventional bacterial and fungal cultures at 37 °C.

Microbial Culture	Ethanol, mg/dL	Ranges of Alcohols’ Concentration, mg/dL			
		1-Propanol	Isobutanol	1-Butanol	Methyl-Butanols
*E. faecalis*, BHI, AA	5–13	0.04–0.10	0–0.01	0.04–0.18	0.02–0.03
*E. faecalis*, BHI, An	3–15	0.05–0.10	0–0.01	0.02–0.18	0.02–0.03
*K. pneumoniae*, BHI, AA	34–59	0.23–1.24	0.01–0.03	0.12–0.22	0.04–0.21
*K. pneumoniae*, BHI, An	33–63	0.19–0.81	0.01–0.03	0.10–0.24	0.03–0.14
*S. aureus*, BHI, AA	10–13	0.10–0.14	0.02–0.03	0.01–0.15	0.02–0.03
*S. aureus*, BHI, An	11–15	0.10–0.18	0.02–0.03	0.01–0.16	0.02–0.04
*C. albicans*, BHI, AA	1–84	0.04–0.15	0.01–0.23	0	0.01–0.91
*C. albicans*, BHI, An	1–86	0.01–0.15	0.08–0.25	0	0.01–1.05
*C. albicans*, SDB, AA	1–964	0.09–0.81	0.03–0.80	0	0.01–1.81
*C. albicans*, SDB, An	1–980	0.06–0.75	0.06–0.63	0	0.10–1.39

**Table 3:** Ranges of alcohols’ concentrations in hDB bacterial and fungal cultures at 25 °C.

hDB Cultures	Alcohols’ Concentration Ranges, mg/dL				
+ Added Glucose	Ethanol, mg/dL	1-Propanol	Isobutanol	1-Butanol	Methyl-Butanols
*E. faecalis*, AA	1–5	0	0	0	0.01–0.12
*E. faecalis*, An	1–7	0–0.01	0	0	0.01–0.18
*K. pneumoniae*, AA	24–52	0.05–0.13	0	0	0.01–0.03
*K. pneumoniae*, An	45–58	0.22–0.79	0	0	0.01–0.03
*S. aureus*, AA	8–55	0.10–0.62	0.01–0.42	0.02–0.27	0.03–0.14
*S. aureus*, An	3–59	0.01–0.57	0.01–0.36	0.08–0.12	0.01–0.14
*C. albicans* ac + 0 g/L glu	1–8	0	0	0	0.01–0.02
*C. albicans* ac + 1 g/L glu	14–53	0.02–0.08	0.01–0.02	0	0.05–0.07
*C. albicans* ac + 3 g/L glu	82–119	0.05–0.14	0.06–0.15	0	0.08–0.14
*C. albicans* ac + 7 g/L glu	179–285	0.09–0.25	0.15–0.41	0	0.12–0.28
*C. albicans* ac + 11 g/L glu	198–472	0.10–0.34	0.17–0.56	0	0.14–0.41
*C. albicans* neu + 0 g/L glu	1–7	0	0	0	0.01–0.02
*C. albicans* neu + 1 g/L glu	28–52	0.02–0.06	0.01–0.07	0	0.04–0.09
*C. albicans* neu + 3 g/L glu	98–125	0.07–0.13	0.08–0.16	0	0.09–0.15
*C. albicans* neu + 7 g/L glu	179–320	0.09–0.22	0.15–0.44	0	0.12–0.30
*C. albicans* neu + 11 g/L glu	199–378	0.16–0.27	0.14–0.44	0	0.13–0.32
*C. albicans* ac + dxtr 5% *w*/*v*	170–386	0.13–0.35	0.19–0.84	0	0.15–0.56

**Table 4:** Successful (E% < ±40%) scores (n) achieved by each microbial model (equations 1–21, Table 1) when applied to samples with ethanol concentration higher than 0.10 g/L (N) from conventional microbial cultures at 37 °C.

Microbial Cultures		EF, BHI/AA	EF, BHI/An	KP, BHI/AA	KP, BHI/An	SA, BHI/AA	SA, BHI/An	CA, BHI/AA	CA, BHI/An	CA, SDB/AA	CA, SDB/An
No	MM										
1	EC1	0/6	0/6	7/8	8/8	0/6	0/6	3/12	9/12	0/12	1/12
2	EC2	0/6	0/6	6/8	8/8	0/6	0/6	4/12	9/12	0/12	1/12
3	EC3	0/6	0/6	6/8	8/8	0/6	0/6	9/12	9/12	0/12	0/12
4	EC4	0/6	0/6	8/8	8/8	0/6	0/6	3/12	8/12	0/12	2/12
5	EC5	0/6	0/6	4/8	8/8	0/6	0/6	0/12	2/12	0/12	0/12
6	EC6	0/6	0/6	8/8	8/8	0/6	0/6	3/12	2/12	0/12	2/12
7	EC7	0/6	0/6	4/8	8/8	0/6	0/6	0/12	0/12	0/12	0/12
8	EF	6/6	6/6	0/8	0/8	6/6	6/6	0/12	1/12	0/12	0/12
9	KP1	0/6	0/6	7/8	6/8	0/6	0/6	7/12	6/12	0/12	0/12
10	KP2	0/6	0/6	1/8	0/8	6/6	4/6	0/12	0/12	0/12	0/12
11	KP3	0/6	0/6	8/8	6/8	0/6	0/6	0/12	0/12	0/12	0/12
12	KP4	6/6	6/6	3/8	0/8	6/6	6/6	0/12	0/12	0/12	0/12
13	SA1	1/6	2/6	6/8	6/8	0/6	0/6	0/12	0/12	0/12	0/12
14	SA2	0/6	0/6	1/8	0/8	6/6	5/6	0/12	0/12	0/12	0/12
15	CA1	0/6	0/6	0/8	0/8	0/6	0/6	10/12	10/12	0/12	2/12
16	CA2	0/6	0/6	0/8	0/8	0/6	0/6	9/12	9/12	1/12	2/12
17	CA3	0/6	0/6	0/8	0/8	0/6	0/6	8/12	9/12	1/12	2/12
18	CA4	0/6	0/6	0/8	0/8	0/6	0/6	0/12	0/12	11/12	10/12
19	CA5	0/6	0/6	0/8	0/8	0/6	0/6	0/12	0/12	11/12	10/12
20	CA6	0/6	0/6	0/8	0/8	0/6	0/6	0/12	0/12	0/12	2/12
21	CA7	0/6	0/6	0/8	0/8	0/6	0/6	0/12	0/12	0/12	2/12

**Table 5:** Successful (E% < ±40%) scores (n) achieved by each microbial model (equations 1–21) when applied to samples with ethanol concentration higher than 0.10 g/L (N) from the hDB cultures at 25 °C.

hDB Culture		*K.p.*, AA	*K.p.,* An	*S.a.*, AA	*S.a.*, An	*C.a.* ac + 1 g/L glu	*C.a.* ac + 3 g/L glu	*C.a.* ac + 7 g/L glu	*C.a.* ac + 11 g/L glu	*C.a.* neu + 1 g/L glu	*C.a.* neu + 3 g/L glu	*C.a.* neu + 7 g/L glu	*C.a.* neu+ 11 g/L glu	*C.a.* ac Dxtr5%*w*/*v*
No	MM													
1	EC1	3/14	0/14	10/10	4/5	5/8	0/8	0/8	0/8	5/8	0/8	0/8	0/8	0/8
2	EC2	3/14	0/14	10/10	4/5	4/8	0/8	0/8	0/8	5/8	0/8	0/8	0/8	0/8
3	EC3	3/14	0/14	10/10	4/5	4/8	0/8	0/8	0/8	5/8	0/8	0/8	0/8	0/8
4	EC4	11/14	14/14	0/10	0/5	4/8	7/8	6/8	1/8	3/8	6/8	6/8	0/8	8/8
5	EC5	13/14	6/14	9/10	4/5	5/8	0/8	0/8	0/8	5/8	0/8	0/8	0/8	0/8
6	EC6	14/14	11/14	10/10	4/5	5/8	0/8	0/8	0/8	8/8	0/8	0/8	0/8	0/8
7	EC7	0/14	0/14	10/10	4/5	0/8	0/8	0/8	0/8	0/8	0/8	0/8	0/8	0/8
8	EF	0/14	0/14	0/10	1/5	1/8	0/8	0/8	0/8	0/8	0/8	0/8	0/8	0/8
9	KP1	0/14	0/14	0/10	1/5	0/8	4/8	6/8	6/8	2/8	5/8	6/8	3/8	7/8
10	KP2	0/14	1/14	0/10	1/5	0/8	0/8	0/8	0/8	0/8	0/8	0/8	0/8	0/8
11	KP3	0/14	0/14	0/10	0/5	0/8	0/8	0/8	0/8	0/8	0/8	0/8	0/8	0/8
12	KP4	0/14	2/14	0/10	1/5	0/8	0/8	0/8	0/8	0/8	0/8	0/8	0/8	0/8
13	SA1	0/14	0/14	0/10	2/5	0/8	0/8	0/8	0/8	0/8	0/8	0/8	0/8	3/8
14	SA2	0/14	2/14	0/10	0/5	0/8	1/8	4/8	0/8	0/8	0/8	5/8	0/8	6/8
15	CA1	8/14	0/14	0/10	0/5	5/8	3/8	0/8	0/8	8/8	4/8	0/8	0/8	0/8
16	CA2	5/14	0/14	0/10	0/5	5/8	6/8	2/8	0/8	4/8	8/8	0/8	0/8	7/8
17	CA3	7/14	0/14	0/10	0/5	5/8	5/8	0/8	0/8	4/8	8/8	0/8	0/8	0/8
18	CA4	0/14	0/14	0/10	0/5	0/8	0/8	0/8	0/8	0/8	0/8	0/8	0/8	0/8
19	CA5	0/14	0/14	0/10	0/5	0/8	2/8	1/8	2/8	0/8	0/8	1/8	3/8	0/8
20	CA6	0/14	0/14	0/10	0/5	0/8	3/8	8/8	8/8	1/8	2/8	8/8	8/8	8/8
21	CA7	0/14	0/14	0/10	0/5	0/8	3/8	1/8	3/8	3/8	1/8	3/8	1/8	0/8

## Data Availability

The data presented in this study are available on request from the corresponding author (privacy and legal issues).
